# Supplementary material for: Modulation of the morphotropic phase boundary for high-performance ductile thermoelectric materials
Source: Nat Commun. 2023 Dec 19;14:8442. doi: 10.1038/s41467-023-44318-4 (PMC10730612; doi:10.1038/s41467-023-44318-4)
Supplement: Supplementary file 1 — Supplementary Information [file 41467_2023_44318_MOESM1_ESM.pdf]

Supplementary materials

Modulation of the morphotropic phase boundary for high-performance ductile thermoelectric materials

Jiasheng Liang#, Jin Liu#, Pengfei Qiu*, Chen Ming, Zhengyang Zhou, Zhiqiang Gao, Kunpeng Zhao*, Lidong Chen, and Xun Shi*

#These two authors contributed equally to this work.
*Corresponding author. Email: qiupf@mail.sic.ac.cn (P.Q.); zkp.1989@sjtu.edu.cn (K.Z.); xshi@mail.sic.ac.cn (X.S.).

This PDF file includes the following sections:

Figs. S1 to S17
Tables S1 to S3
References

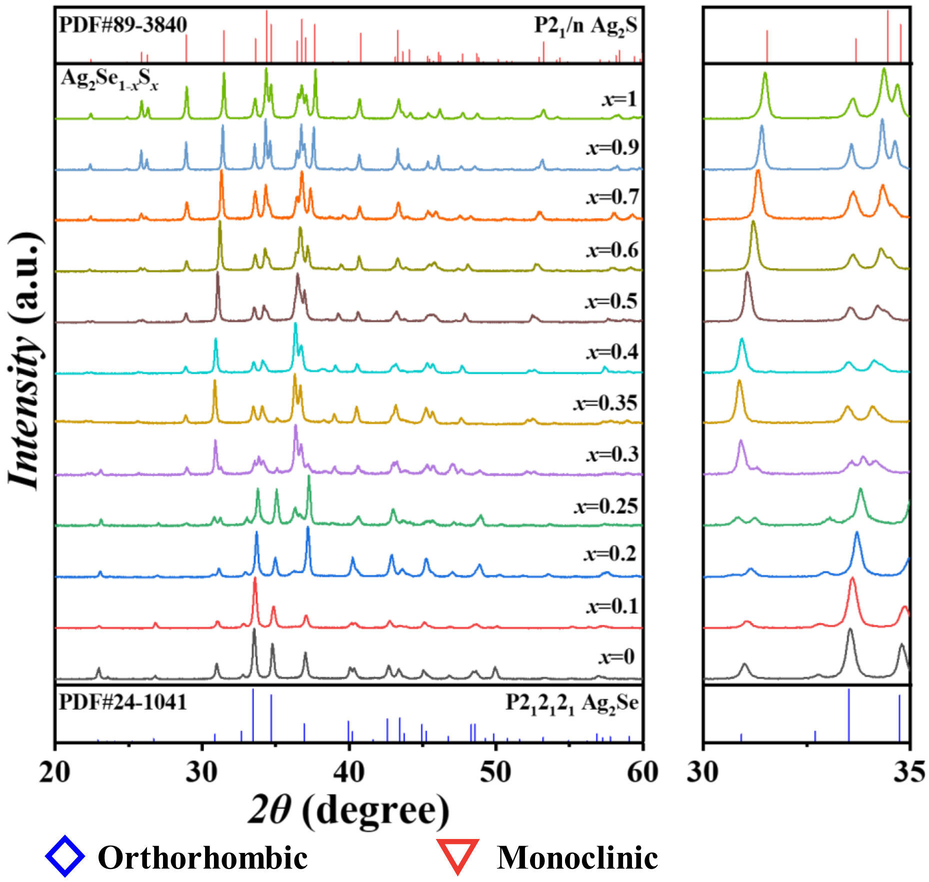

Fig. S1| Room-temperature XRD patterns of the as-prepared bulk $Ag_2Se_{1-x}S_x$ pseudobinary compounds. The right panel shows the magnification at 2θ = 30°–35°.

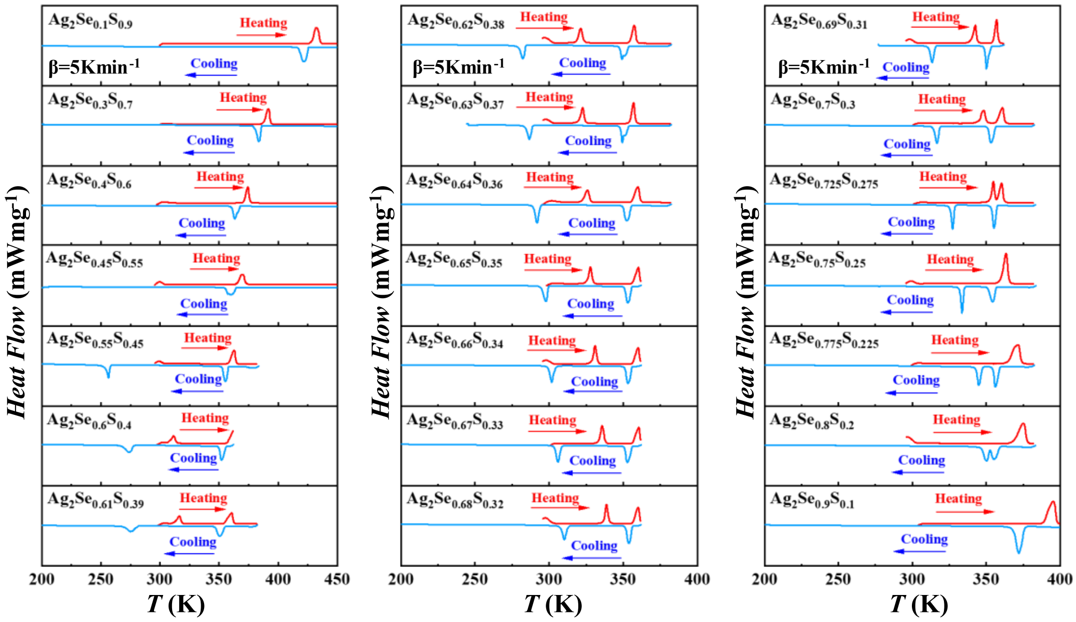

Fig. S2| Heat flow curves of $Ag_2Se_{1-x}S_x$ pseudobinary compounds. The blue lines are obtained in the cooling process, and the red lines are obtained in the heating process. The heating/cooling rate is 5 K min^{-1}.

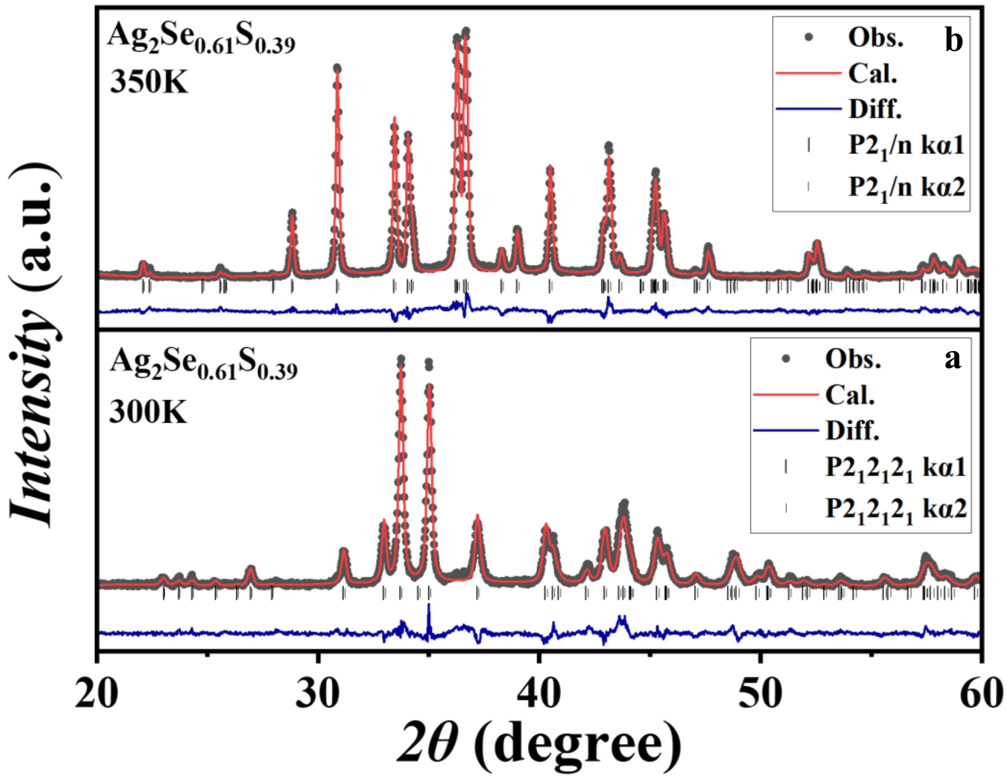

Fig. S3| Rietveld refinement of the powder X-ray diffraction patterns of $Ag_2Se_{0.61}S_{0.39}$ collected at (a) 300 K and (b) 350 K. The black circles represent the measured point intensities. The red lines represent the intensities calculated from the assigned structures. The blue lines show the difference between the experimental and calculated intensities. The black vertical bars indicate Bragg positions.

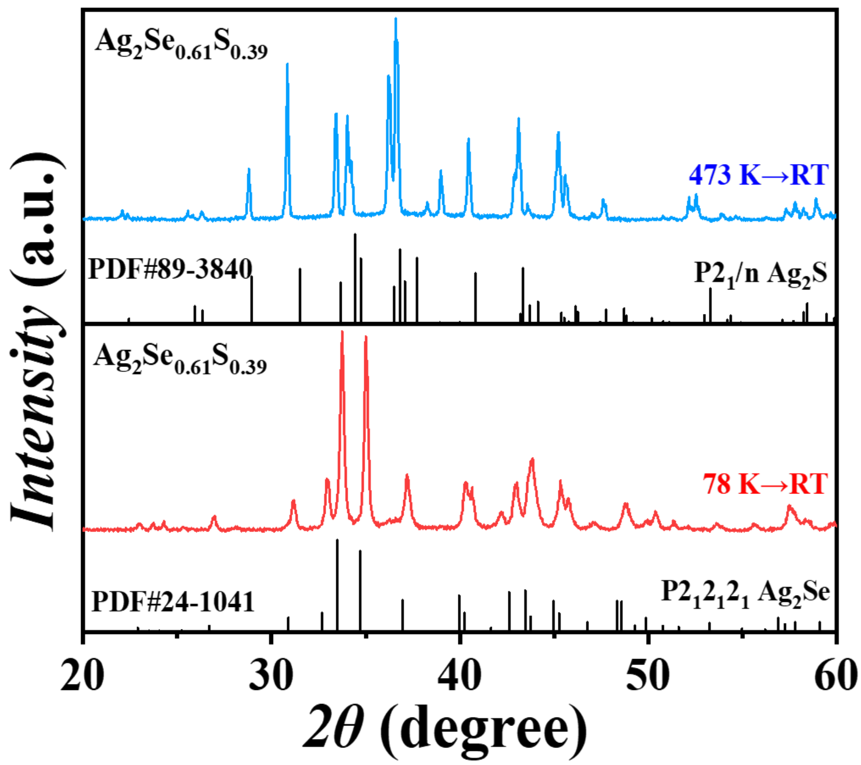

Fig. S4| Room-temperature XRD diffraction patterns for $Ag_2Se_{0.61}S_{0.39}$ after heating (78 K→RT) and cooling (473 K→RT).

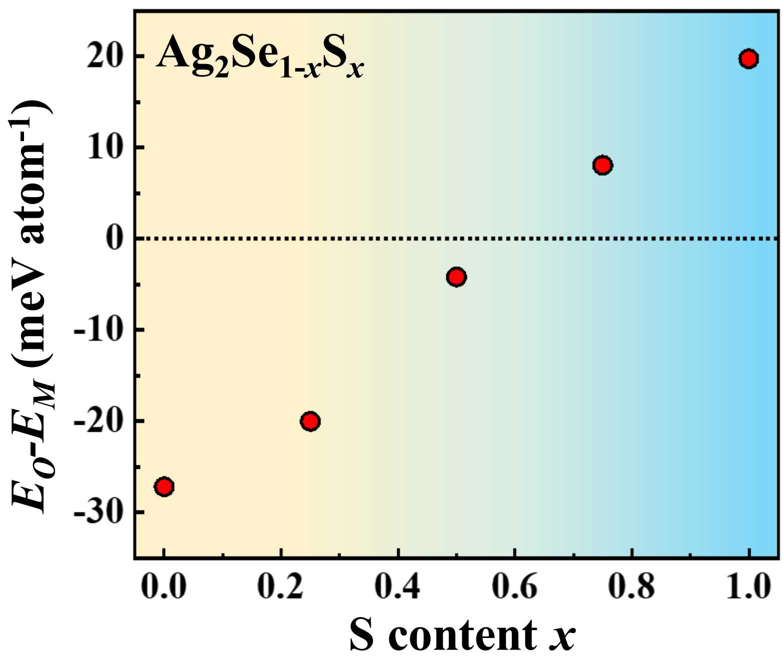

Fig. S5| Energy difference between orthorhombic and monoclinic $Ag_2Se_{1-x}S_x$ pseudobinary compounds. E_O is the orthorhombic structure, and E_M is the monoclinic structure.

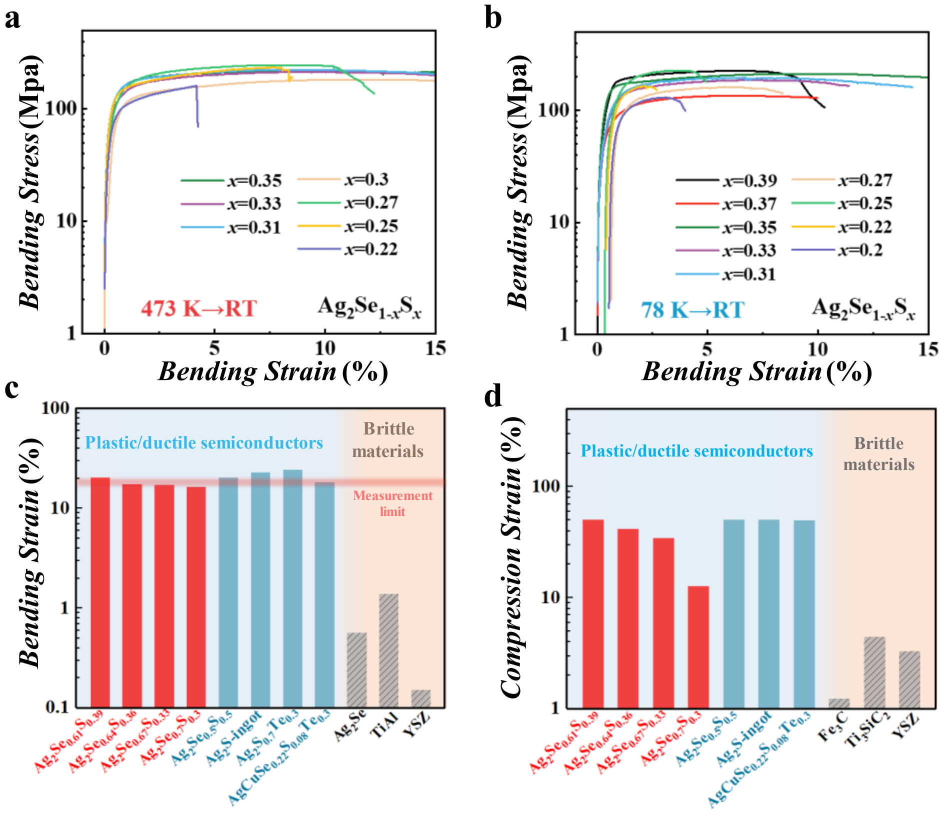

Fig. S6| Mechanical properties of $Ag_2Se_{1-x}S_x$. Engineering stress–strain curves of the three-point bending test performed on orthorhombic $Ag_2Se_{1-x}S_x$ pseudobinary compounds with chemical compositions in the vicinity of MPB after experiencing different thermal histories. The data in (**a**) are for the samples cooled from 473 K to room temperature. The data in (**b**) are for the samples naturally heated from liquid N_2 (78 K) to the ambient atmosphere. (**c–d**) Comparisons of the maximum engineering stress strain of the three-point bending test and compressing test performed on ductile orthorhombic $Ag_2Se_{1-x}S_x$ specimens developed in this work and some ductile inorganic semiconductors reported previously. The data of some brittle materials are included for comparison. The data are taken from Refs. [1–8].

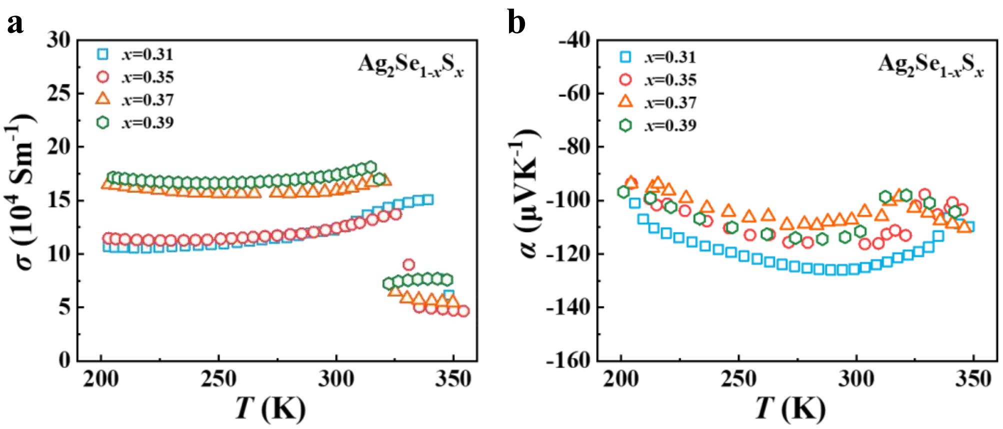

Fig. S7| Electrical conductivity and Seebeck coefficient. Temperature dependences of the (**a**) electrical conductivity (σ) and (**b**) Seebeck coefficient (α) for $Ag_2Se_{1-x}S_x$ ($0.3 < x < 0.4$).

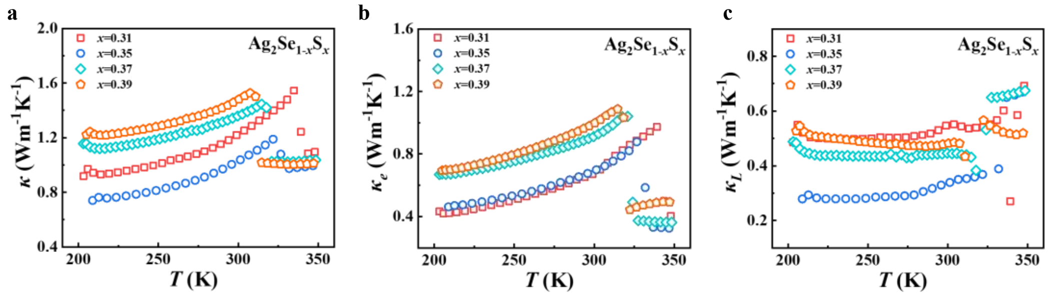

Fig. S8| Thermal conductivity. Temperature dependences of the (**a**) thermal conductivity (κ), (**b**) electron lattice thermal conductivity (κ_e), and (**c**) lattice thermal conductivity (κ_L) for $Ag_2Se_{1-x}S_x$ ($0.3 < x < 0.4$).

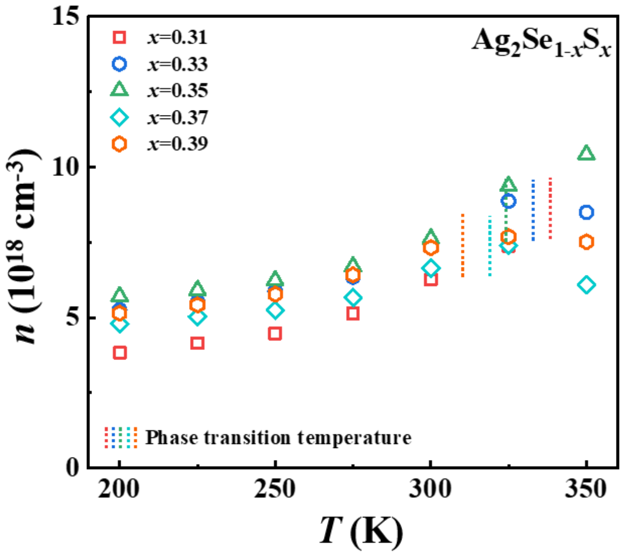

Fig. S9| Temperature dependence of carrier concentration (n) for $Ag_2Se_{1-x}S_x$ ($0.3 < x < 0.4$).

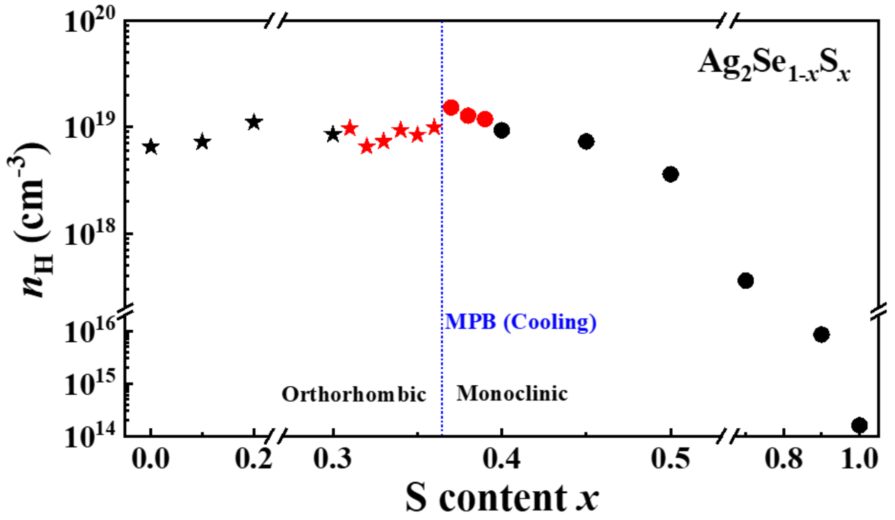

Fig. S10| Room-temperature carrier concentration (n) as a function of S content x for $Ag_2Se_{1-x}S_x$.

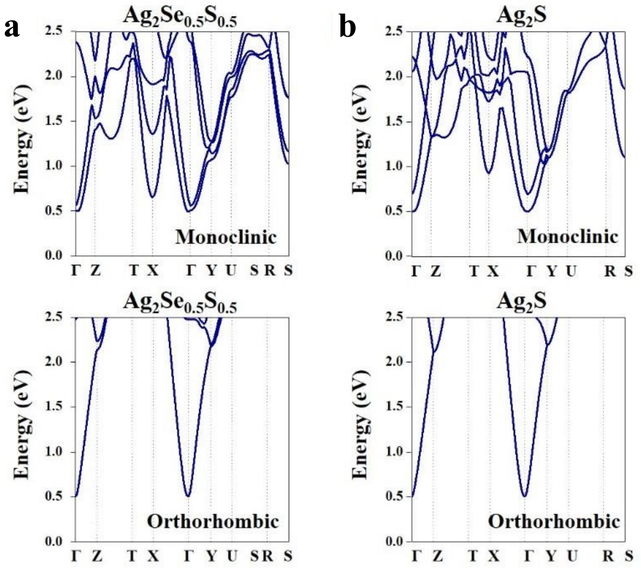

Fig. S11| Band structures of (a) $Ag_2Se_{0.5}S_{0.5}$, (b) Ag_2S, and (c) Ag_2Se crystalizing in orthorhombic and monoclinic structures.

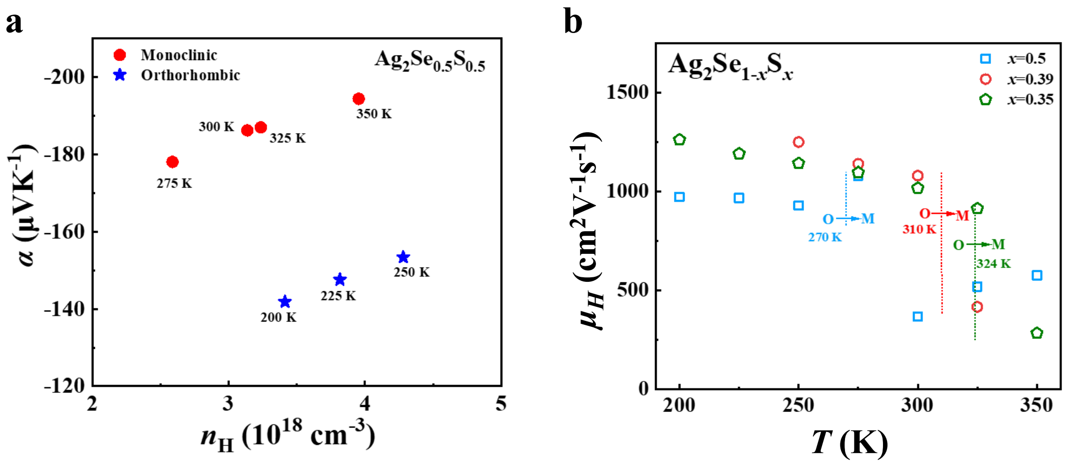

Fig. S12| Seebeck coefficient and carrier mobility. (**a**) Seebeck coefficient (α) as a function of the Hall concentration (n_H) for $Ag_2Se_{0.5}S_{0.5}$. The circles represent the monoclinic structure. The pentagrams represent the orthorhombic structure. (**b**) Temperature dependence of the Hall carrier mobility (μ_H). Obvious μ_H reduction can be observed after the orthorhombic–monoclinic phase transition.

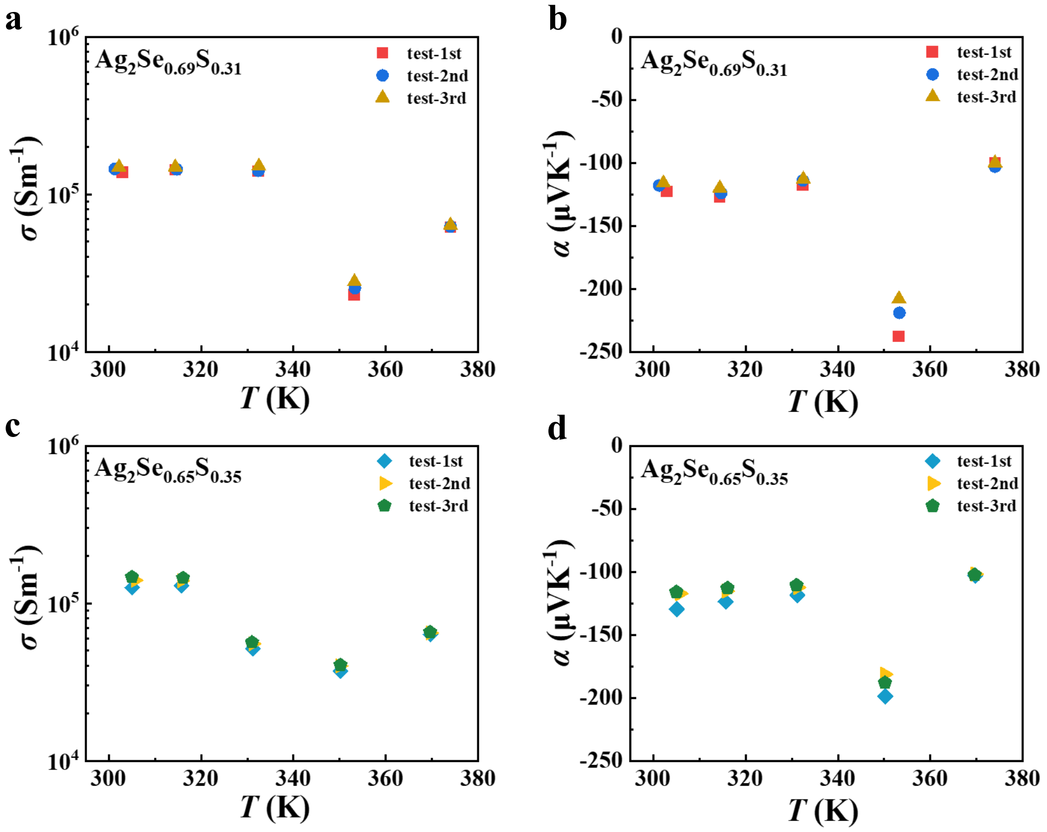

Fig. S13| Cyclic measurements of electrical transport properties between 300 K and 370 K. (**a**) electrical conductivity (σ) of $Ag_2Se_{0.69}S_{0.31}$. (**b**) Seebeck coefficient (α) of $Ag_2Se_{0.69}S_{0.31}$. (**c**) electrical conductivity (σ) of $Ag_2Se_{0.65}S_{0.35}$. (**d**) Seebeck coefficient (α) of $Ag_2Se_{0.65}S_{0.35}$.

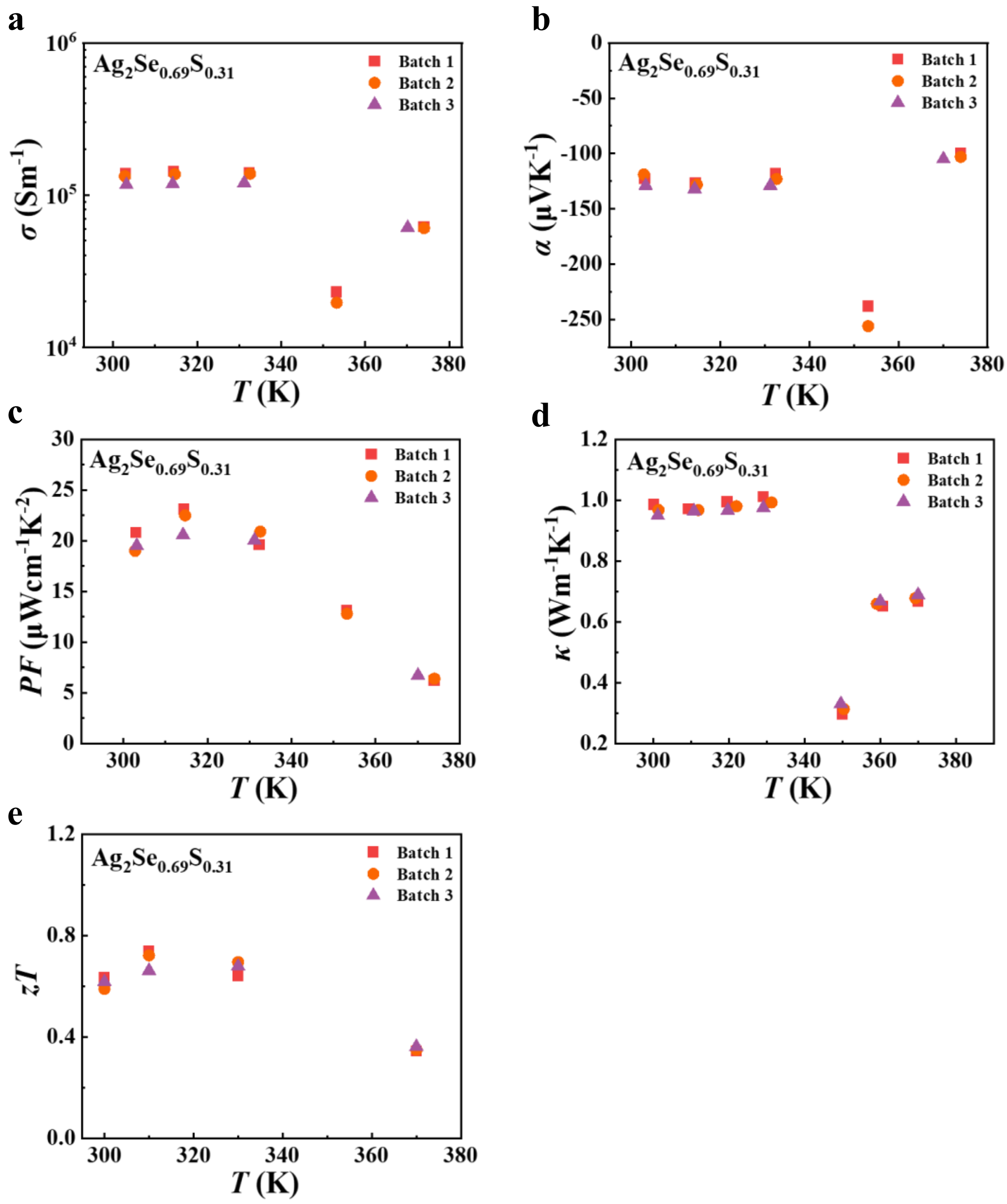

Fig. S14| Thermoelectric properties of three batches of $Ag_2Se_{0.69}S_{0.31}$. (**a**) electrical conductivity (σ), (**b**) Seebeck coefficient (α), (**c**) Power factor (PF), (**d**) thermal conductivity (κ), and (**e**) figure-of-merit value (zT) of $Ag_2Se_{0.69}S_{0.31}$.

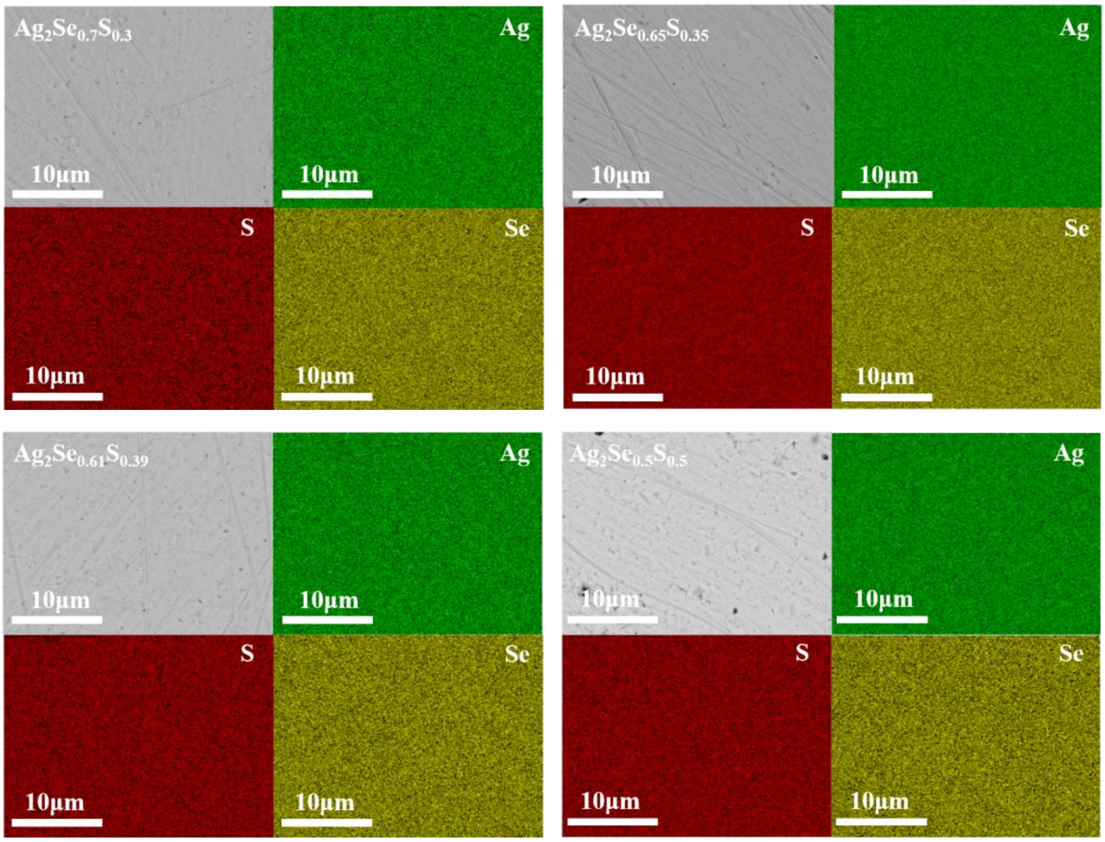

Fig. S15| Elemental distribution of $Ag_2Se_{1-x}S_x$. Backscatter electron (AsB) image and elemental energy dispersive spectroscopy (EDS) mappings of $Ag_2Se_{1-x}S_x$ (x = 0.3, 0.35, 0.39, and 0.5). The magnification is 10000×.

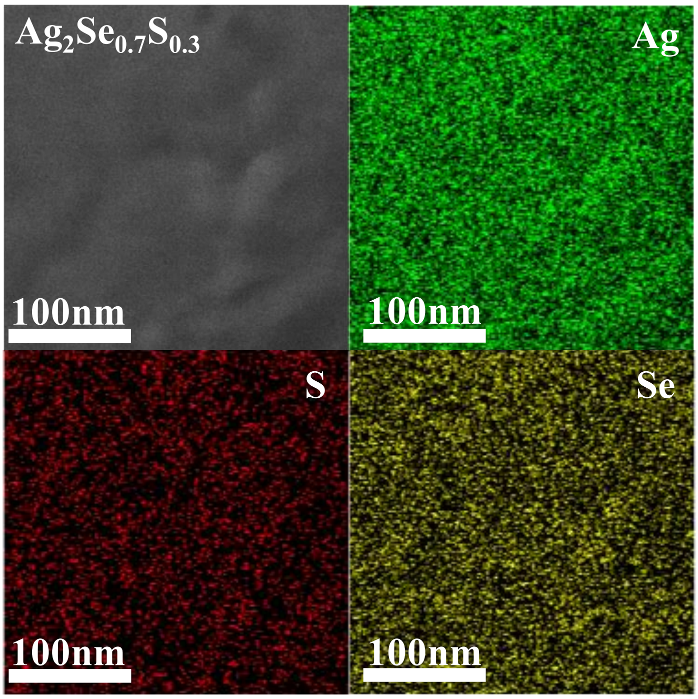

Fig. S16| Elemental energy dispersive spectroscopy (EDS) mappings of $Ag_2Se_{0.7}S_{0.3}$ characterized by TEM equipment. The specimen was fabricated by using the ultrathin section technique. Due to the intrinsic ductility of the material, the specimen is not very flat, which is responsible for the contrast shown in the figures.

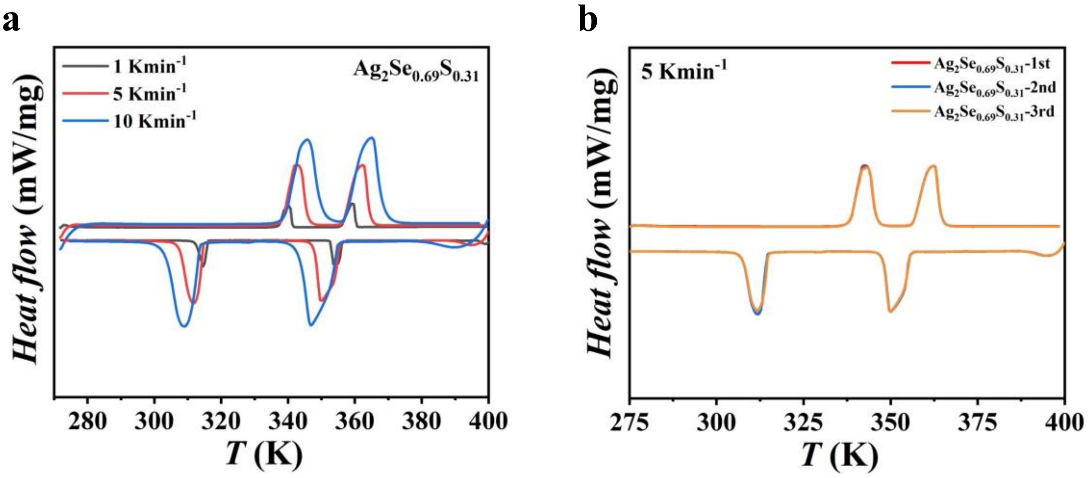

Fig. S17| Heat flow curves of $Ag_2Se_{0.69}S_{0.31}$. (**a**) Heat flow curves of $Ag_2Se_{0.69}S_{0.31}$ with different heating/cooling rates (1 $Kmin^{-1}$, 5 $Kmin^{-1}$, and 10 $Kmin^{-1}$). (**b**) Cyclic measurements of the heat flow curves of $Ag_2Se_{0.69}S_{0.31}$ with a heating/cooling rate of 1 K min^{-1}.

Table S1| Refined residual parameters for $Ag_2Se_{0.61}S_{0.39}$ at 300 K and 350 K.

T **(K)**	**Space group**	***R* (obs)**	***wR* (obs)**	***R* (all)**	***wR* (all)**	R_p	wR_p
300	$P2_12_12_1$	3.88%	4.22%	3.95%	4.23%	8.91%	11.44%
350	$P2_1/n$	4.97%	5.45%	5.98%	5.77%	5.96%	7.60%

Table S2| Calculated electron effective masses of Ag_2S, $Ag_2S_{0.5}Se_{0.5}$, and Ag_2Se in monoclinic and orthorhombic structures. The unit of the effective mass is m_e. M represents the monoclinic structure, and O represents the orthorhombic structure.

	Phase	Γ→X	Γ→Y	Γ→Z	Average
Ag_2S	M	0.54	0.88	0.45	0.62
	O	0.21	0.15	0.18	0.18
$Ag_2S_{0.5}Se_{0.5}$	M	0.29	0.74	0.29	0.44
	O	0.19	0.13	0.14	0.15
Ag_2Se	M	0.27	0.25	0.16	0.23
	O	0.17	0.12	0.14	0.14

Table S3| Room-temperature thermoelectric properties of $Ag_2Se_{1-x}S_x$ samples with compositions near the MPB.

Composition	S (μV·K^{-1})	σ (S·m^{-1})	κ (W·m^{-1}·K^{-1})	κ_e	κ_L	PF (μW·cm^{-1}·K^{-2})	zT
$Ag_2Se_{0.6}S_{0.4}$	-99	6.4×10^4	0.60	0.37	0.23	6.2	0.31
$Ag_2Se_{0.61}S_{0.39}$	-83	6.3×10^4	1.05	0.37	0.68	4.4	0.15
$Ag_2Se_{0.62}S_{0.38}$	-83	8.6×10^4	0.94	0.51	0.43	5.9	0.22
$Ag_2Se_{0.63}S_{0.37}$	-81	8.6×10^4	1.01	0.51	0.50	5.6	0.17
$Ag_2Se_{0.64}S_{0.36}$	-128	1.1×10^5	1.04	0.60	0.44	18.1	0.52
$Ag_2Se_{0.65}S_{0.35}$	-111	1.4×10^5	1.10	0.77	0.32	16.8	0.46
$Ag_2Se_{0.66}S_{0.34}$	-117	1.4×10^5	1.06	0.79	0.27	19.2	0.54
$Ag_2Se_{0.67}S_{0.33}$	-116	1.4×10^5	1.04	0.81	0.23	19.5	0.56
$Ag_2Se_{0.68}S_{0.32}$	-119	1.3×10^5	0.98	0.70	0.28	17.8	0.54
$Ag_2Se_{0.69}S_{0.31}$	-126	1.4×10^5	1.08	0.76	0.32	22.0	0.61
$Ag_2Se_{0.7}S_{0.3}$	-120	1.5×10^5	1.08	0.85	0.23	21.8	0.60

References

1. Liang, J. et al. Flexible thermoelectrics: from silver chalcogenides to full-inorganic devices. *Energy Environ. Sci.* **12**, 2983-2990 (2019).
2. Yang, Q. *et al.* Flexible thermoelectrics based on ductile semiconductors. *Science* **377**, 854-858 (2022).
3. Shi, X. *et al.* Room-temperature ductile inorganic semiconductor. *Nat. Mater.* **17**, 421-426 (2018).
4. Yang, S. *et al.* Ductile $Ag_{20}S_7Te_3$ with excellent shape-conformability and high thermoelectric performance. *Adv. Mater.* **33**, 2007681 (2021).
5. Gnanamoorthy, R., Mutoh, Y. & Mizuhara, Y. Flexural strength of gamma base titanium aluminides at room and elevated temperatures. *Mater. Sci. Eng.* **197**, 69-77 (1995).
6. Lankford, J., Page, R. A. & Rabenberg, L. Deformation mechanisms in yttria-stabilized zirconia. *J. Mater. Sci.* **23**, 4144-4156 (1988).
7. Shabanova, I. N. & Trapeznikov, V. A. A study of the electronic structure of Fe_3C, Fe_3Al and Fe_3Si by x-ray photoelectron spectroscopy. *J. Electron Spectrosc.* **6**, 297-307 (1975).
8. Sun, Z. M., Zhang, Z. F., Hashimoto, H. & Abe, T. Ternary compound Ti_3SiC_2: Part II. Deformation and fracture behavior at different temperatures. *Mater. Trans.* **43**, 432-435 (2002).
